# Cytochrome 4Z1 Expression Connotes Unfavorable Prognosis in Ovarian Cancers

**DOI:** 10.3390/medicina58091263

**Published:** 2022-09-13

**Authors:** Yousef M. Al-saraireh, Fatemah O. F. O. Alshammari, Anas O. Satari, Yanal S. Al-mahdy, Ghadeer H. Almuhaisen, Omar H. Abu-azzam, Ala N. Uwais, Seham M. Abufraijeh, Ahlam M. Al-Kharabsheh, Sa’ed M. Al-dalain, Aiman Al-Qtaitat, Fatima Al-Tarawneh, Jehad M. Al Shuneigat, Sameeh A. Al-Sarayreh

**Affiliations:** 1 Department of Pharmacology, Faculty of Medicine, Mutah University, P.O. Box 7, Al-Karak 61710, Jordan; 2 Department of Medical Lab Technology, Faculty of Health Sciences, The Public Authority for Applied Education and Training, Shuwaikh 15432, Kuwait; 3 Faculty of Medicine, Mutah University, P.O. Box 7, Al-Karak 61710, Jordan; 4 Department of Microbiology and Pathology, Faculty of Medicine, Mutah University, P.O. Box 7, Al-Karak 61710, Jordan; 5 Department of Obstetrics and Gynecology, Faculty of Medicine, Mutah University, P.O. Box 7, Al-Karak 61710, Jordan; 6 Department of Anatomy and Histology, Faculty of Medicine, Mutah University, P.O. Box 7, Al-Karak 61710, Jordan; 7 Faculty of Dentistry, Zarqa University, Zarqa 13110, Jordan; 8 Department of Allied Medical Sciences, Faculty of Al-Karak, Al-Balqa Applied University, P.O. Box 7, Al-Karak 61710, Jordan; 9 Department of Biochemistry and Molecular Biology, Faculty of Medicine, Mutah University, P.O. Box 7, Al-Karak 61710, Jordan

**Keywords:** cancer, Cytochrome P450, Cytochrome 4Z1, Immunohistochemistry, Ovarian cancer

## Abstract

*Background and Objective:* Ovarian cancer is a leading cause of death in females. Since its treatment is challenging and causes severe side effects, novel therapies are urgently needed. One of the potential enzymes implicated in the progression of cancers is Cytochrome 4Z1 (CYP4Z1). Its expression in ovarian cancer remains unknown. Therefore, the current study aims to assess CYP4Z1 expression in different subtypes of ovarian cancers. *Materials and Methods*: Immunohistochemistry was used to characterize CYP4Z1 expression in 192 cases of ovarian cancers along with eight normal ovarian tissues. The enzyme’s association with various clinicopathological characteristics and survival was determined. *Results:* CYP4Z1 was strongly expressed in 79% of ovarian cancers, compared to negative expression in normal ovarian samples. Importantly, significantly high CYP4Z1 expres-sion was determined in patients with advanced-stage cancer and a high depth of invasion (*p* < 0.05). Surprisingly, CYP4Z1 expression was significantly associated with a low patient survival rate. Univariate analysis revealed that patient survival was strongly associated with CYP4Z1 expression, tumor stage, depth of invasion, and lymph node metastasis (*p* < 0.05). Multivariate analysis showed that only CYP4Z1 expression was significantly associated with patient survival (*p* < 0.05). *Conclusions:* CYP4Z1 expression is correlated with shorter patient survival and has been identified as an independent indicator of a poor prognosis for ovarian cancer patients.

## 1. Introduction

Ovarian cancers are a heterogeneous group of neoplasms that originate in the ovaries or other related areas of the fallopian tube and peritoneum [1]. According to the GLOBOCAN estimates of cancer incidence and mortality worldwide, ovarian cancer is a leading type of cancer in females and is ranked as the third most common gynecological cancer [2]. The epidemiology of ovarian cancer shows that it is also associated with poor prognosis and a high mortality rate. In other words, almost half of females with ovarian cancer die within five years after diagnosis. This low relative survival is mainly attributed to inadequate screening programs and late patient presentation at diagnosis [3]. Ovarian cancers can be divided into two main types: epithelial and non-epithelial, with epithelial being more dominant [4]. Under these two classifications are many types of histological subtypes that differ in various aspects, including pathology and clinical features [5]. Regarding the etiology behind ovarian cancer, specific causes are relatively unknown. Nonetheless, some risk factors have been found to contribute to its development. Among these factors are family history, the prevalence of BRCA1 and BRCA2 mutations, HNPCC syndrome (Lynch II syndrome), a prophylactic oophorectomy, nulliparity and infertility, and environmental, dietary, and, finally, host factors [6]. Ovarian cancer patients are commonly diagnosed by using a combination of a serum cancer antigen-125 (CA-125) test and transvaginal ultrasound. However, the positive predictive value for detection of invasive ovarian cancer with this combination is nearly low. Although other biochemical markers for screening or detection of ovarian cancer have been developed, none of these have shown potential for clinical use so far [7]. Despite modern advances in cancer therapy, clinical management of this disease is challenging and remains difficult. Therefore, there is an urgent need to search for novel biomarkers and drug targets for the development of new therapies for ovarian cancers.

Several studies have highlighted the importance of the selective expression of orphan cytochrome P450s (CYPs) in cancers for therapeutic purposes [8,9,10]. For instance, the development of CYP19 inhibitors for breast cancer therapy represented the first successful approach targeting CYP enzymes in cancer treatment. For ovarian cancer, many CYPs were found to be overexpressed compared to normal ovarian tissues. These include but are not limited to CYP2J2, CYP2S1, CYP1B1, CYP2U1, and CYP3A. Despite some of these enzymes not being as specific for ovarian cancer, the presence of these enzymes may pave the way for the discovery of novel CYP-targeted therapies [10,11]. The aberrant expression of these enzymes in cancers has attracted researchers’ interest, particularly CYP4Z1. This unique enzyme was selectively found in mammary tissues, with an absence of expression in other normal tissues [12]. Interestingly, CYP4ZI was strongly overexpressed in breast cancer [12,13] and, more recently, was also determined in many cancers of the colon, prostate, bladder, ovary, and cervix [14,15,16,17,18]. Importantly, CYP4ZI expression was more frequently found in patients with advanced stages of the disease and connoted a worse prognosis [13,14,16,17]. CYP4Z1 was abnormally translocated at breast cancer cell membranes and stimulated the formation of anti-CYP4Z1 autoantibodies in breast cancer patient sera in comparison with none in the control group [19,20]. Such translocation was conditionally regulated by treatment with glucocorticoids and progesterone and inhibited by treatment with mifepristone (steroid receptor blocker) [21].

As CYP4Z1 is considered an orphan enzyme, a limited number of studies have investigated its substrate spectrum and catalytic features [22,23,24,25,26]. Using the CYP4Z1 homology model and different mutants of recombinant CYP4Z1, different key residues for substrate recognition were determined, including Arg487, Ser113, Asn381, Asn381, Ser383, and Ser222 [22,23]. By screening several luminogenic substrates by utilizing CYP4Z1-containing enzyme bags, luciferin benzyl ether was determined as the best substrate [23]. These recent advances led to the identification of 1-benzylimidazole as the first selective mechanism-based inhibitor for CYP4Z1, showing a potent inhibitory effect against CYP4Z1 with a minimal inhibitory profile against other CYPs [25]. Additionally, using systematic virtual screening, another selective potent CYP4Z1 inhibitor (Compound 9) was also developed, showing high selectivity and nanomolar affinity to the CYP4Z1 enzyme [26]. All of these recent developments may encourage the discovery of novel anti-cancer targeted therapies.

Since existing ovarian cancer treatment strategies are limited and challenging, novel treatment approaches, especially for metastatic diseases, are urgently needed. Due to the latest success with CYP inhibitors as anti-cancer therapy, such as aromatase (CYP19) inhibitors [27], the potential to develop novel therapies may be offered by selective CYP4Z1 expression in certain cancers. In a preliminary study utilizing a small number of samples, CYP4Z1 expression was successfully characterized in certain cancers, including ovarian cancer [28]. However, there are no data on CYP4Z1 expression in a large cohort of different pathological subtypes of ovarian cancers. Therefore, the current study aims to investigate the frequency of CYP4Z1 in various pathological subtypes of ovarian cancers and its association with histopathological features, as well as prognosis.

## 2. Materials and Methods

### 2.1. Tissue Specimens

Prior to the start of the study, the necessity for informed consent was waived because informed consent for the use of archived paraffin ovarian cancer samples is exempted by the Institutional Review and Ethics Committee of the Faculty of Medicine, University of Mutah (Reference No. 6012021 date: 20 January 2021). The study was designed and performed according to the guidelines of the Declaration of Helsinki. The current study included subjects from King Abdullah University Hospital and King Hussein Medical Hospital, Jordan from 2013 to 2020. Ovarian cancer tissue samples and normal ovarian tissue samples were fixed in formalin and embedded in paraffin. After that, they were cut into 5-µm-thick tissue sections and then stained with hematoxylin and eosin for routine diagnostic evaluation. The inclusion criteria for this study were being a female diagnosed with ovarian cancer and aged 18–80 years (192 cases). The study also included eight cases of healthy normal ovarian samples as a control group. Any case that had radiotherapy or chemotherapy before the surgery was excluded from the study. The panel of ovarian tumors consisted of 86 cases of serous adenocarcinoma, 48 cases of serous papillary adenocarcinoma, 22 cases of mucinous adenocarcinoma, 14 cases of mucinous papillary adenocarcinoma, eight cases of endometrioid adenocarcinoma, and 14 cases of Krukenberg tumor. Patients’ data on histological grade, clinical stage, TNM (Tumor, Nodes, and Metastases) classification, age, and tumor histology were obtained from medical records. In this study, survival data were only available for 100 cases, which ranged from 15 to 60 months (median 58 months). Overall survival was measured from the date of surgical intervention to the date of the last follow-up or death. All patient-related data and information were kept anonymous and confidential.

### 2.2. Immunohistochemistry

Tissue slides were firstly dewaxed in xylene and then rehydrated in a series of decreasing gradients of alcohol and, finally in distilled water. Quenching endogenous peroxidase activity was achieved by placing the slides in 3% hydrogen peroxide for ten minutes. Then, the slides were washed in PBS, and antigen retrieval was carried out by microwaving at 650 W in 0.01 M citrate buffer for 20 minutes. After antigen retrieval, non-specific binding sites were blocked using 2.5% normal goat serum. For primary antibodies, the slides were incubated overnight at 4 °C in 5 μg/mL with a CYP4Z1 polyclonal rabbit antibody (NBP1-91817, Novus Biologicals, Centennial, CO, USA). The specificity of the aforementioned antibody towards CYP4Z1 was confirmed by Western blotting using whole lysates of engineered CYP4Z1 cells. To further confirm the antibody’s specificity, the CYP4Z1 antibody was incubated with CYP4Z1 blocking protein (H00199974-P01, Novus Biologicals, Centennial, CO, USA) for 60 minutes at room temperature. The resulting mixture was then applied to the tissues in place of the primary antibody. The staining density was compared between the slides treated with blocked antibody and the slides incubated with only the primary antibody. The following day, tissue sections were incubated with ImmPRESS (peroxidase) goat anti-rabbit IgG polymer for 30 minutes at room temperature. Immunoreactivity was developed using 3,39-diaminobenzidine chromogen substrate (DAB), and Harris’ hematoxylin was chosen for counterstaining. After dehydration with an increasing gradient of alcohol and xylene, the slides were mounted with coverslips using DPX. A breast cancer tissue sample was used as a positive control. For the negative control, the tissue slide was incubated with normal goat serum instead of the primary antibody under the same conditions. Immunoreactivity was evaluated using a Leica DMRB microscope. Images were taken using a JVC video camera and then digitally processed.

### 2.3. Scoring

The evaluation of the slides was carried out by two independent pathologists using the Allred scoring system [29,30]. This system uses a combination of the percentage and the intensity of the staining. To assess the percentage of membranous- or cytoplasmic-stained cells, a score of 0–5 was used. Negative expression in the tissue section corresponded to a score of 0. Tissue sections showing expression levels of less than 1% had a score of 1. Tissue sections were given a score of 2 if they showed expression levels between 1–10%. A score of 3 was given to tissue sections showing expression levels between 11 and 33%. Tissue sections showing expression levels between 34 and 66% were given a score of 4. Lastly, a score of 5 was given to tissue sections showing expression levels of more than 67%. As for staining intensity, it was scored from 0–3; negative staining was given a score of 0, weak staining was given a score of 1, moderate staining was given a score of 2, and strong staining was given a score of 3 (Figure S1). The final score was equal to the summation of both the staining intensity score and the frequency of the staining score. Thus, the generated score had eight possible numbers of the score. A score of less than or equal to 2 was labeled as negative for CYP4Z1 expression, and a score from 3–8 was labeled as positive for CYP4Z1 expression.

### 2.4. Statistical Analysis

Data were analyzed using SPSS version 25.(IBM, Armonk, NY, USA) Categorical variables were expressed in frequencies, and differences between variables were measured using the multinomial goodness-of-fit test. A Kaplan–Meier curve was used to measure patients’ overall survival, and a log rank test was used to measure statistical significance. In addition, the CYP4Z1 prognostic value was evaluated by using univariate and multivariate Cox regression at confidence limits (Cl) of 95%. A *p*-value of less than 0.05 was deemed significant.

## 3. Results

### 3.1. Baseline Demographics and Clinicopathological Characteristics

Table 1 shows the baseline demographics and the clinicopathological characteristics of the 200 female participants. The study consisted of 192 tissue cases of different ovarian cancer subtypes, and eight cases were adjacent to normal ovarian tissues. The estimated average age of the participants was 48.2 ± 10.6 years. Of the participants, 61.5% (123 cases) were under the age of 50, while 38.5% (77 cases) were aged more than 50 years. The majority of the patients were at tumor grade III (65.3%, 115 cases), while 20.5% (36 cases) and 14.2% (25 cases) were at tumor grade II and tumor grade I, respectively. Regarding the tumor histological stage, most of the patients were at tumor stage I (84.7%, 149 cases), while tumor stage II and tumor stage III comprised 8% (14 cases) and 7.4% (13 cases), respectively. Moreover, about 84.1% of patients (148 cases) had a tumor limited to one or both ovaries (T1), while 11.9% (21 cases) had a tumor in the ovaries with pelvic implants (T2), and only 4% (seven cases) had a tumor in the ovaries with confirmed peritoneal metastasis outside the pelvis (T3). Additionally, only 7.4% (13 cases) of patients presented with lymph node metastasis, and the rest of the patients (92.6%, 163 cases) were free from lymph node metastasis.

### 3.2. Prevalence of CYP4Z1 Expression and Its Relation to Clinicopathological Features

**Figure 1:**
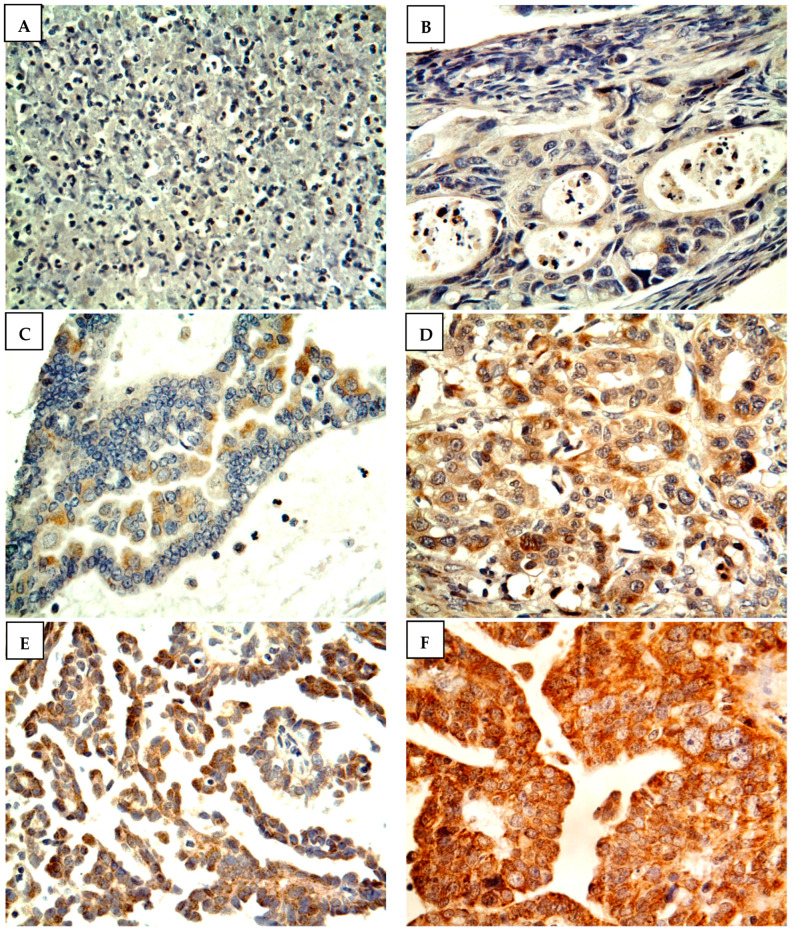
Immunostaining and scoring guide of CYP4Z1 expression in ovarian cancer. The expression was observed as either membranous or cytoplasmic staining. (**A**) Score “0” reveals totally negative expression, (**B**) score “1” shows expression of less than 1% of cells, (**C**) score “2” shows expression levels between 1 and 10% of cells, (**D**) score “3” shows expression between 11 and 33% of cells, (**E**) score “4” illustrates expression between 34 and 66% of cells, and (**F**) score “5” shows expression levels of more than 67% of cells. Magnification: ×400.

**Figure 2:**
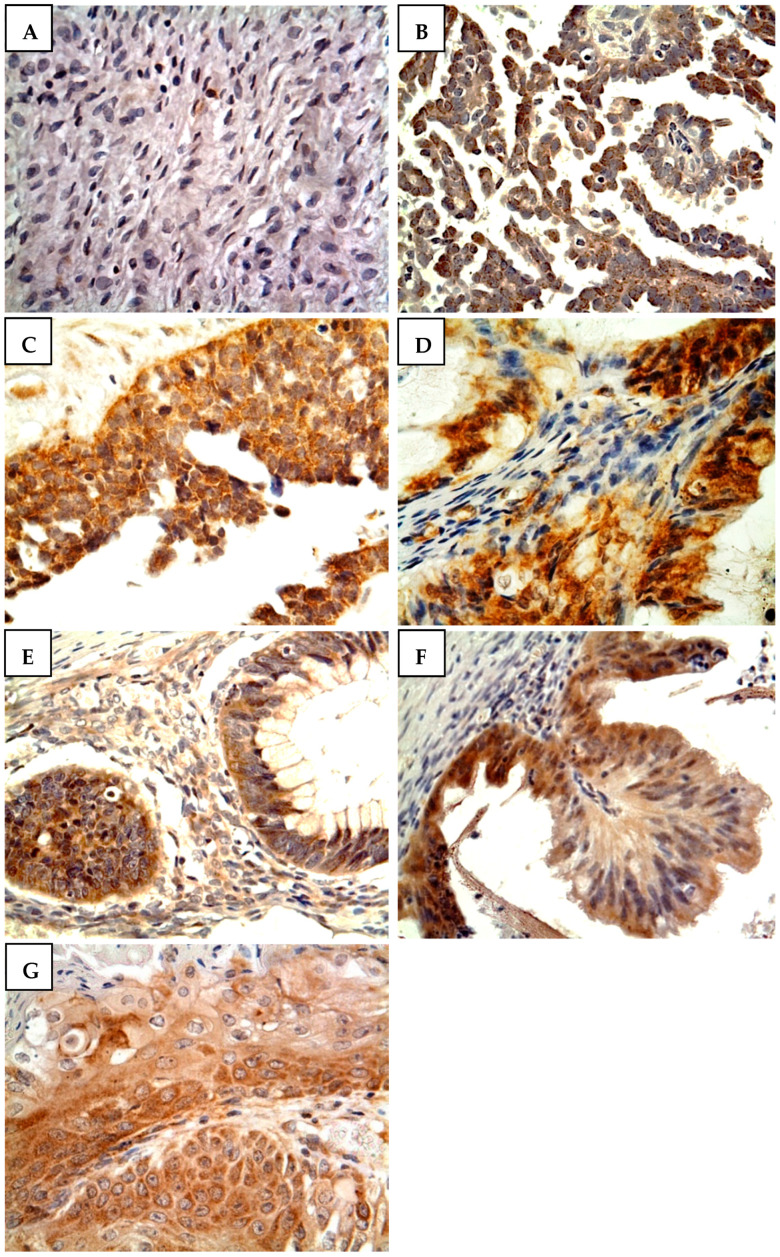
CYP4Z1 expression in different subtypes of ovarian cancer. Tumors were classified according to histopathological subtypes: (**A**) normal ovarian tissue, (**B**) serous papillary adenocarcinoma, (**C**) serous adenocarcinoma, (**D**) mucinous papillary adenocarcinoma, (**E**) mucinous adenocarcinoma, (**F**) Krukenberg tumor, and (**G**) endometrioid adenocarcinoma. Magnification: ×400.

Figure 1 shows the scoring criteria for CYP4Z1 expression. CYP4Z1 expression was detected in 79% of patients (158 cases), where it was displayed in the cytoplasm or membranes of cells. Only 12.5% of normal ovarian tissues showed positive CYP4Z1 expression, while others showed negative expression (Figure 2). Importantly, CYP4Z1 expression was validated by using appropriate negative and positive controls and inhibition of immunostaining with the CYP4Z1-blocking antibody. There was no observable immunostaining in negative controls, while strong immunoreactivity was exhibited in positive controls (breast cancer tissues). In addition, immunostaining was not identified in ovarian cancer specimens treated with a mixture of blocked CYP4Z1 antibodies (Figure S2).

Data analysis revealed a significant association between CYP4Z1 expression and pathological subtype, histological stage, and tumor depth of invasion (*p* < 0.05; Table 1). There was a marked difference in the CYP4Z1 expression between normal tissue samples and different histopathological subtypes of ovarian cancer. Furthermore, CYP4Z1 expression was prevalent in all ovarian cancer pathological subtypes. However, papillary adenocarcinoma tumors of either serous or mucinous subtypes showed discriminable expression of CYP4Z1 from other histopathological subtypes (91.7%, 44 cases and 100%, 14 cases, respectively). Moreover, there were high levels of CYP4Z1 expression in patients with stage III (100%, 13 cases) and stage II tumors (100%, 14 cases) compared to those with stage I tumors (79.2%, 118 cases). Of the positive patients, a high frequency of CYP4Z1 expression was found in patients with confirmed peritoneal metastasis outside the pelvis (T3, 100%, seven cases) and confirmed metastasis in the pelvis (T2, 100%, 21 cases) compared to that in patients with tumors confined only to the ovaries (T1, 79.1%, 117 cases). However, no significant correlations were detected between CYP4Z1 expression and age, histological grade, and lymph node metastasis.

### 3.3. The Correlation between CYP4Z1 Expression and Prognosis in Ovarian Cancer

**Figure 3:**
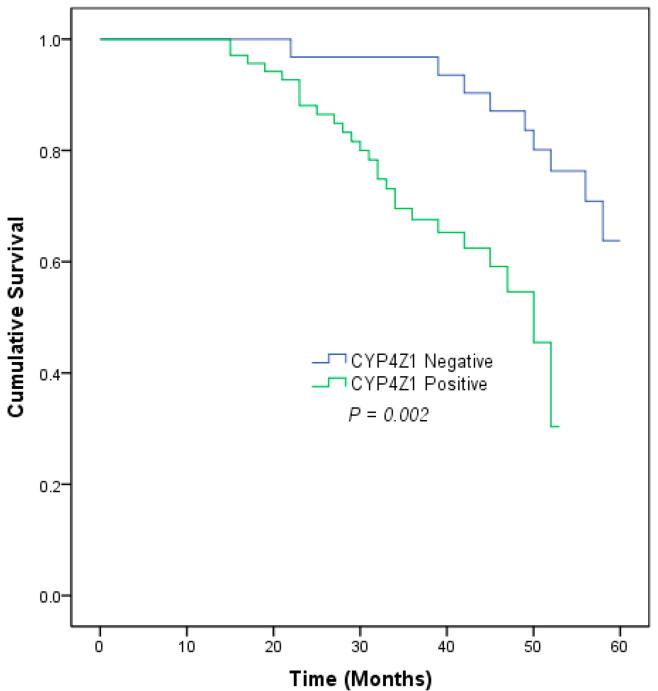
Kaplan–Meier survival curve of ovarian cancer patients based on CYP4Z1 expression.

Survival data were only available for 100 cases of ovarian cancers. Patients were classified according to their CYP4Z1 expression into two groups: positive expression (69%, 69 cases) and negative expression (31%, 31 cases). The patients’ survival rate was analyzed with the Kaplan–Meier curve, and significance was calculated in a log rank test. The analysis revealed that there was a significant association between CYP4Z1 expression and ovarian cancer patients’ survival rate (*p* = 0.002). Positive CYP4Z1 patients had a poor survival rate (62.3%, mean = 42.9 ± 1.5 months) compared to negative CYP4Z1 patients (71.0%, mean = 55.6 ± 1.5 months) (Figure 3). In a univariate Cox regression analysis, CYP4Z1 expression, histological stage, tumor depth of invasion, and lymph node metastasis had a significant influence on the overall survival rate (*p* < 0.05; Table 2). To confirm these results and eliminate any bias that might have been caused by the univariate analysis, a multivariate Cox regression was conducted. The results showed that CYP4Z1 expression was the only independent predictor of poor prognosis of ovarian cancer patients (*p* = 0.01; HR= 1.177, 95% CI = 1.040–1.332) (Table 2).

## 4. Discussion

The global increase in ovarian cancer mortality and morbidity has made it a significant health problem. This type of cancer is considered very aggressive and has a relatively poor prognosis compared with other types of cancers [2]. The current treatment regimens for this disease are not effective and cause severe toxicity. Almost all ovarian cancer patients suffer from at least a single episode of chemotherapy-related toxicity after receiving multiple cycles of combinatory chemotherapeutic regimens [31]. Consequently, finding new biomarkers and therapeutic targets that are useful in the therapy of ovarian cancer is necessary. This is an exciting field of study, as there are new and ongoing prospects for research that could reveal novel features of CYP4Z1 in the development and treatment of cancer. In an initial screening that used a limited number of tumor samples, we identified selective expression of CYP4Z1 in various types of cancers, including ovarian cancer [28]. This observation has encouraged further research in order to deeply investigate CYP4Z1 expression in a larger panel of different types of ovarian cancers.

As only one study has explored CYP4Z1 expression in a small cohort of ovarian cancers [16], the current study has identified CYP4Z1 expression in a wide range of different histopathological types of ovarian cancers. The results showed that 79% of the ovarian cancers investigated had CYP4Z1 expression, and the expression was confined to tumor cells. Normal ovarian tissues showed almost negative expression for CYP4Z1. These results are in agreement with our initial screening [28] and a previous study showing a similar fashion of expression [16]. Furthermore, our findings are in line with the Human Protein Atlas data on CYP4Z1 transcription profiling in ovarian cancer. When compared to normal ovarian tissues, ovarian cancers have high CYP4Z1 mRNA levels [32]. Importantly, this trend in the differential expression of CYP4Z1 was identified in many cancer types, including ovarian cancer [13,14,16,17,18,28]. This differential in CYP4Z1 expression was able to allow discrimination between benign, primary, and metastatic breast, colon, and ovarian cancers [14,16,28].

In the current study, the CYP4Z1 enzyme’s role as a clinicopathological marker in ovarian cancer was assessed. There were significant associations between CYP4Z1 expression and pathological subtype, tumor stage, and tumor depth of invasion. A high frequency of CYP4Z1 expression was exhibited in papillary adenocarcinomas (serous and mucinous) compared to that in other pathological subtypes. Moreover, CYP4Z1 was more frequently expressed in patient tumors at an advanced stage of disease compared to patient tumors in the early stages of the disease. This trend was identified by other studies showing elevated CYP4Z1 expression in advanced stages as opposed to early stages of disease [12,13,17,28]. Additionally, CYP4Z1 expression was found to be greater in tumors with confirmed metastasis in the pelvis (T2) and confirmed peritoneal metastasis outside the pelvis (T3) than in tumors confined only to the ovaries (T1). Interestingly, CYP4Z1 expression was associated with a poor survival rate of ovarian cancer patients and identified as an independent factor for overall survival. Such a significant association has been reported in many studies linking CYP4Z1 expression with poor patient survival and connecting it to aggressive characteristics of cancers such as breast, colon, prostate, cervical, and ovarian cancers [13,14,15,16,17]. Our results imply that CYP4Z1 has a possible role in ovarian cancer progression and metastasis.

As there are no functional studies assessing the mechanistic role of CYP4Z1 in the progression of ovarian cancer, several studies have linked CYP4Z1 with cancer development in general [21,24,33,34,35,36]. By using in vitro and in vivo models, CYP4Z1 expression was found to significantly enhance tumor proliferation, tumor angiogenesis, and tumor metastasis. Furthermore, CYP4Z1 overexpression promoted the expression of the vascular endothelial growth factor A (VEGF-A) and decreased the expression of the tissue inhibitor of metalloproteinases 2 (TIMP-2) in cancer cells compared to control cells [33]. All of these were also accompanied by the production of high levels of 20-hydroxyeicosatetraenoic acid (20-HETE) and reduced levels of lauric and myristic acids [24,33]. Such changes in levels of fatty acids were reported by an earlier study where CYP4Z1 converted lauric and myristic acids into various monohydroxylated products and metabolized archidonic acid into 20-HETE [24,33]. However, in the latest report, it was revealed that CYP4Z1 has an epoxygenase activity that transforms arachidonate into 14, 15-epoxyeicosatrienoate (14, 15-EET) [34]. Importantly, this ligand was found to enhance tumor growth and angiogenesis [37]. Further investigation into the mechanisms by which CYP4Z1 contributes to tumorigenesis revealed that expression of the pseudogenes CYP4Z1-3′UTRs and CYP4Z2P synergistically increased tumor angiogenesis in breast cancer partly via the activation of the PI3K/Akt and ERK1/2 pathways [36]. Furthermore, it was revealed that expression of CYP4Z1 promoted breast cancer cells’ stemness and resistance to tamoxifen [35]. Altogether, these findings provide significant evidence that CYP4Z1 may contribute to tumor progression and metastasis.

## 5. Conclusions

A distinct expression of CYP4Z1 was characterized in all pathological subtypes of ovarian cancers in comparison with the lack of expression in normal ovarian tissues. Significantly high CYP4Z1 expression was found in patients with advanced stages of disease and tumor depth of invasion. Significantly, CYP4Z1 expression was correlated with shorter survival and connoted a poor prognosis for ovarian cancer patients. Overall, the CYP4Z1 enzyme could be used as a biomarker and potential target for the discovery and development of novel therapies for ovarian cancer.

## Figures and Tables

**Table 1:** Baseline demographic and clinicopathological features of ovarian cancer patients.

	CYP4Z1 Expression		
Characteristic	Negative *n* = 42 (21%)	Positive *n* = 158 (79%)	*p* Value
**Age:**			
<50 (*n* = 123, 61.5%)	23 (18.7%)	100 (81.3%)	0.373
≥50 (*n* = 77, 38.5%)	19 (24.7%)	58 (75.3%)	
**Pathology subtype:**			
Serous adenocarcinoma (*n* = 86,43%)	20 (23.3%)	66 (76.7%)	0.005
Serous papillary adenocarcinoma (*n* = 48, 24%)	4 (8.3%)	44 (91.7%)	
Mucinous adenocarcinoma (*n* = 22, 11%)	5 (22.7%)	17 (77.3%)	
Mucinous papillary adenocarcinoma (*n* = 14, 7%)	0 (0%)	14 (100%)	
Endometrioid adenocarcinoma (*n* = 8, 4%)	3 (37.5%)	5 (62.5%)	
Krukenberg tumor (*n* = 14, 7%)	3 (21.4%)	11 (78.6%)	
Normal (*n* = 8, 4%)	7 (87.5%)	1 (12.5%)	
**Histological grade**:			
I (*n* = 25, 14.2%)	4 (16%)	21 (84%)	0.082
II (*n* = 36, 20.5%)	2 (5.6%)	34 (94.4%)	
III (*n* = 115, 65.3%)	25 (21.7%)	90 (78.3%)	
**Histological stage:**			
I (*n* = 149, 84.7%)	31 (20.8%)	118 (79.2%)	0.033
II (*n* = 14, 8%)	0 (0%)	14 (100%)	
III (*n* = 13, 7.4%)	0 (0%)	13 (100%)	
**Tumor depth of invasion:**			
T1 (*n* = 148, 84.1%)	31 (20.9%)	117 (79.1%)	0.028
T2 (*n* = 21, 11.9%)	0 (0%)	21 (100%)	
T3 (*n* = 7, 4%)	0 (0%)	7 (100%)	
**Lymph node metastasis:**			
Negative (*n* = 163, 92.6%)	31 (19%)	132 (81%)	0.073
Positive (*n* = 13, 7.4%)	0 (0%)	13 (100%)	

**Table 2:** Univariate and multivariate analyses of prognostic variables associated with ovarian cancer patients’ survival.

Prognostic Variable	Univariate			Multivariate		
	HR	95%Cl	*p*-Value	HR	95%Cl	*p*-Value
Age	0.756	0.383–1.494	0.421	0.631	0.297–1.340	0.231
Pathology subtype	1.081	0.824–1.417	0.575	1.153	0.851–1.561	0.358
Histological grade	1.255	0.770–2.045	0.361	1.288	0.766–2.168	0.340
Histological stage	2.461	1.577–3.840	0.001	3.061	0.652–14.371	0.156
Tumor depth of invasion	2.088	1.227–3.555	0.007	0.487	0.125–1.902	0.301
Lymph node metastasis	7.599	2.928–19.719	0.001	2.803	0.360–21.839	0.325
CYP4Z1 expression	1.155	1.034–1.299	0.010	1.177	1.040–1.332	0.010

## Data Availability

The data presented in this study are available on request from the corresponding author. The data are not publicly available due to privacy and ethical concerns.

## Supplementary Materials

The following supporting information can be downloaded at: https://www.mdpi.com/article/10.3390/medicina58091263/s1, Figure S1. CYP4Z1 antibody staining intensity. Negative staining, score 0 (blue arrow); weak staining, score 1 (red arrow); moderate staining, score 2 (yellow arrow) and strong staining, score 3 (green arrow). Magnification (×400). Figure S2: CYP4Z1 expression in different types of experimental controls. (A) No CYP4Z1 immunoreactivity was determined in ovarian cancer tissue incubated with normal goat serum instead of CYP4Z1primary antibody (negative control), (B) High CYP4Z1expression was displayed in ovarian cancer tissue incubated with CYP4Z1 primary antibody, (C) Weak to no CYP4Z1 expression was seen in ovarian cancer tissue incubated with mixture of primary antibody and blocking peptide, (D) High CYP4Z1 expression was exhibited in breast cancer tissue incubated with CYP4Z1 primary antibody (positive control) and (E) weak CYP4Z1 expression was detected in breast cancer tissue incubated with mixture of primary antibody and blocking peptide. Magnification (×400).
