# Supplementary material for: Cytochrome 4Z1 Expression Connotes Unfavorable Prognosis in Ovarian Cancers
Source: Medicina (Kaunas). 2022 Sep 13;58(9):1263. doi: 10.3390/medicina58091263 (PMC9502355; doi:10.3390/medicina58091263)
Supplement: Supplementary file 1 [file medicina-58-01263-s001.zip › medicina-1906400-supplementary.pdf]

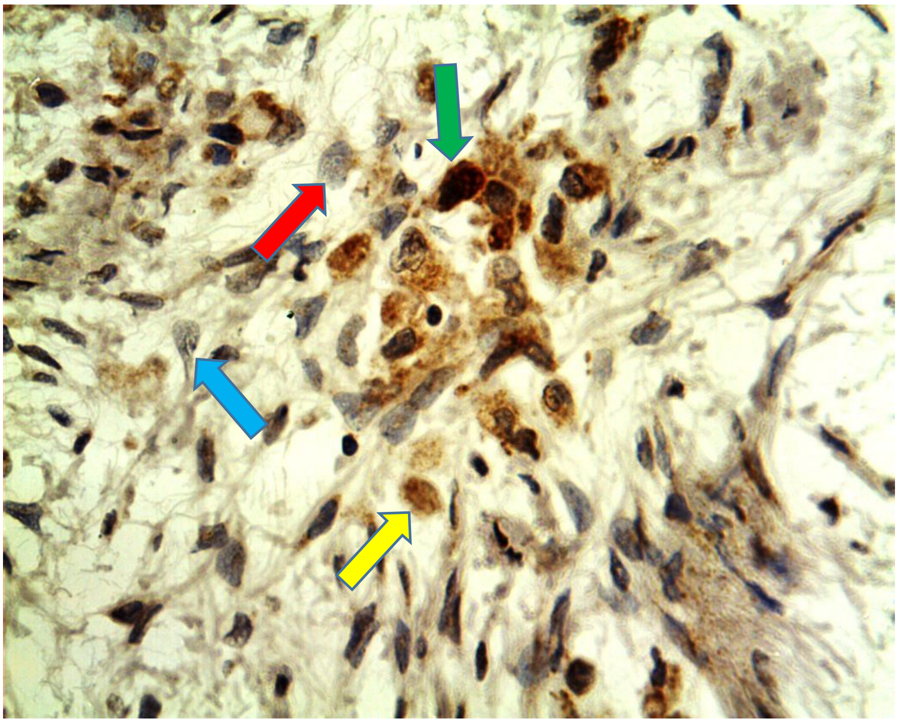

Figure S1. CYP4Z1 antibody staining intensity. Negative staining, score 0 (blue arrow); weak staining, score 1 (red arrow); moderate staining, score 2 (yellow arrow) and strong staining, score 3 (green arrow). Magnification (x400).

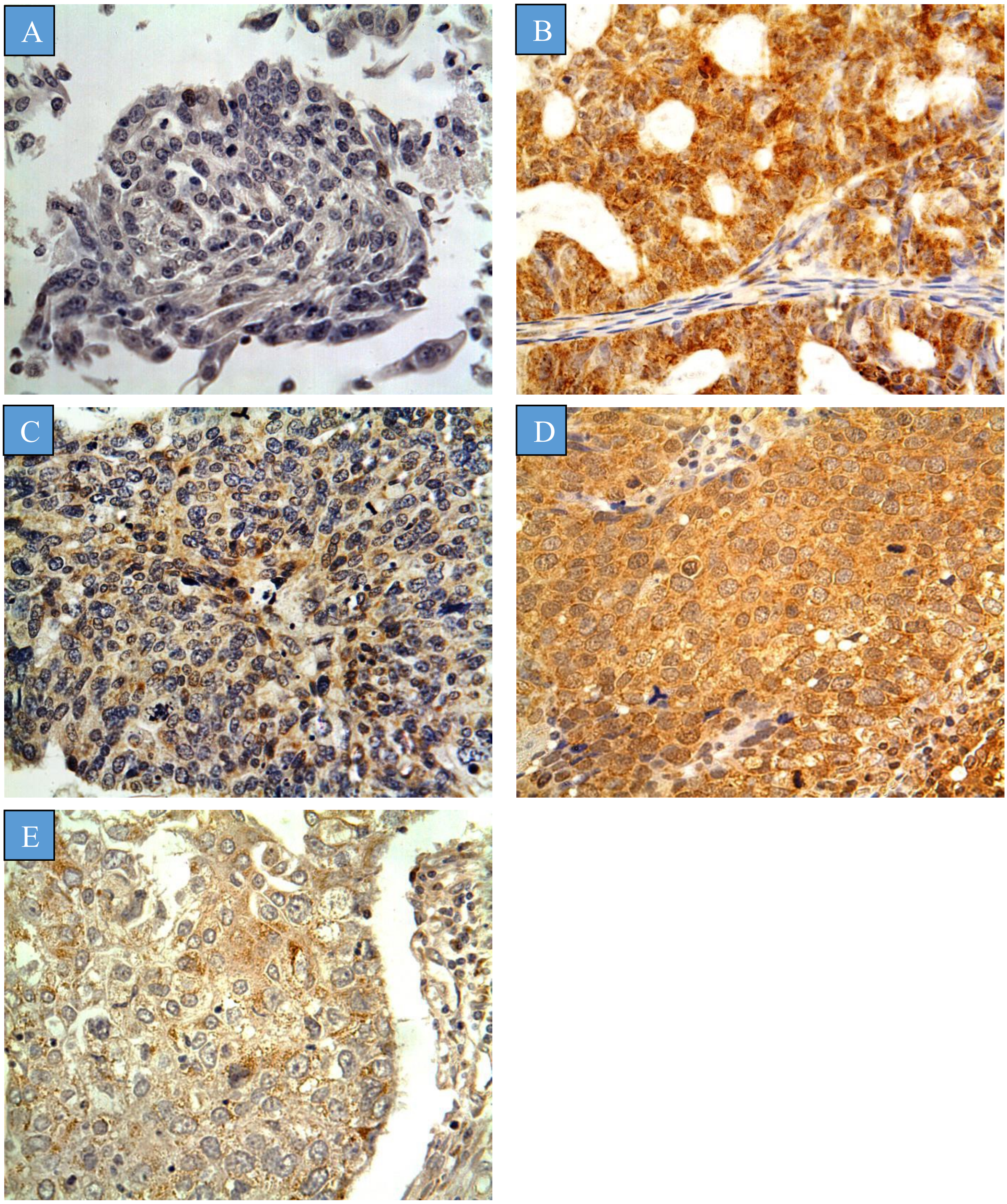

Figure S2. CYP4Z1 expression in different types of experimental controls. (A) No CYP4Z1 immunoreactivity was determined in ovarian cancer tissue incubated with normal goat serum instead of CYP4Z1primary antibody (negative control), (B) High CYP4Z1expression was displayed in ovarian cancer tissue incubated with CYP4Z1primary antibody , (C) Weak to no CYP4Z1 expression was seen in ovarian cancer tissue incubated with mixture of primary antibody and blocking peptide, (D) High CYP4Z1expression was exhibited in breast cancer tissue incubated with CYP4Z1primary antibody (positive control) and (E) weak CYP4Z1 expression was detected in breast cancer tissue incubated with mixture of primary antibody and blocking peptide. Magnification (x400).
